# Design and Characterization of Baricitinib Incorporated PLA 3D Printed Pills by Fused Deposition Modeling: An Oral Pill for Treating Alopecia Areata

**DOI:** 10.3390/polym15081825

**Published:** 2023-04-08

**Authors:** Mohammed Muqtader Ahmed, Farhat Fatima, Aisha Alnami, Mohammad Alsenaidy, Alhussain H. Aodah, Mohammed F. Aldawsari, Bjad Almutairy, Md. Khalid Anwer, Mohammed Jafar

**Affiliations:** 1 Department of Pharmaceutics, College of Pharmacy, Prince Sattam Bin Abdulaziz University, P.O. Box 173, Al-Kharj 11942, Saudi Arabia; 2 Department of Pharmaceutical Chemistry, Faculty of Pharmacy, King Abdulaziz University, Jeddah 21589, Saudi Arabia; 3 Department of Pharmaceutics, College of Pharmacy, King Saud University, P.O. Box 2457, Riyadh 11451, Saudi Arabia; 4 Department of Pharmaceutics, College of Clinical Pharmacy, Imam Abdulrahman Bin Faisal University, P.O. Box 1982, Dammam 34212, Saudi Arabia

**Keywords:** Baricitinib, PLA, FDM, FTIR, DSC, in vitro release and kinetics

## Abstract

This study aimed to develop three-dimensional (3D) baricitinib (BAB) pills using polylactic acid (PLA) by fused deposition modeling. Two strengths of BAB (2 and 4% *w*/*v*) were dissolved into the (1:1) PEG-400 individually, diluting it with a solvent blend of acetone and ethanol (27.8:18:2) followed by soaking the unprocessed 200 cm~6157.94 mg PLA filament in the solvent blend acetone—ethanol. FTIR spectrums of the 3DP1 and 3DP2 filaments calculated and recognized drug encapsulation in PLA. Herein, 3D-printed pills showed the amorphousness of infused BAB in the filament, as indicated by DSC thermograms. Fabricated pills shaped like doughnuts increased the surface area and drug diffusion. The releases from 3DP1 and 3DP2 were found to be 43.76 ± 3.34% and 59.14 ± 4.54% for 24 h. The improved dissolution in 3DP2 could be due to the higher loading of BAB due to higher concentration. Both pills followed Korsmeyer–Peppas’ order of drug release. BAB is a novel JAK inhibitor that U.S. FDA has recently approved to treat alopecia areata (AA). Therefore, the proposed 3D printed tablets can be easily fabricated with FDM technology and effectively used in various acute and chronic conditions as personalized medicine at an economical cost.

## 1. Introduction

*Alopecia areata* (AA) is an autoimmune disorder often resulting from an unpredictable immune system that attacks hair follicles and causes hair loss. An epidemiological study reflected that the incidence of AA is approximately 1 in 1000 people globally. It affects roughly 147 million people worldwide. In the United States alone, there are 6.8 million people who suffer from AA [1,2]. This condition is more common among African Americans than Asians and people of white descent. Children and adults may develop AA at a similar rate in all genders. Statistically, it has been reported that AA has approximately a 2% chance of incidence during one’s lifetime [3]. Hair loss could be from any body part, but AA usually affects the head and face. It has been fairly represented as a coin-sized patch of hair fall [4]; therefore, AA is usually characterized by patchy baldness, which could be on the scalp, beard area, eyebrows, eyelashes, armpits, inside nose, or ears. The leading cause of AA is unknown; however, it is widely attributed to the attack of white blood cells (autoimmune system) on the hair follicles, leading to the shrinking and the slowing down of hair growth followed by inflammation.

Hair follicles are immune-privileged sites that can tolerate antigens without eliciting inflammatory immune responses. Its complex structure provides immunity against the body’s immune systems [2]. These sites have low primary histocompatibility complex classes I and II expression with well-suppressed natural killer cells. The disorientation of these complex systems or defects in natural killer cells results in the loss of immune privilege and ultimately causes AA. Both genetic and non-genetic factors can influence AA [5].

Moreover, AA is genetically transferred from one generation of family members to the next, so the chances of the dominance of this condition are one in five people with such a disease [6]. A family history of AA also represents other autoimmune disorders, such as atopy conditions. Occasionally, AA is also triggered by viral infections (influenza), which cause excess production of interferons-γ, collapsing the immune system [7]. AA therapy includes corticosteroids and anti-inflammatory medications, including minoxidil and anthralin [8], which suppress the immune system. The dosages of these drugs are mostly administered through local injections, topical ointment applications, or oral pills. Despite multiple therapies for AA, it cannot be entirely cured as most patients experience future episodes of hair loss. Corticosteroids can be directly administered at the site of the hair loss patch to stimulate hair growth. This process may take up to eight weeks, and the treatment is continued and repeated every four to six weeks until complete regrowth is achieved [9]. Although oral corticosteroids are preferred in case of extensive hair loss, long-term treatment is not recommended due to lethal side effects. Topical application of minoxidil lengthens the growth phase of hair follicles and stimulates hair growth. Subsequently, it takes about 12 weeks for new hair growth. However, this treatment method is ineffective in patients with severe AA [10].

A new form of AA therapy—investigational treatment of AA by Janus kinase (JAK) inhibitors—has shown promising results. The repurposing of JAK inhibitors in AA has been well-established in pre-clinical studies. The U.S. FDA recently approved using baricitinib (BAB) oral tablets to treat adult patients with severe AA [11,12]. BAB is a JAK inhibitor that blocks enzymes’ activity, interfering with the functioning of inflammation pathways. Genome-wide association studies (GWAS) and functional immunological studies have identified CD8+ cells that cause apoptosis in cells, an NKG2D receptor that recognizes stress-induced surface ligands, and T lymphocyte and thymocyte that fight cancer. These CD8+ NKG2D + T cells are the major effectors of AA, which promote hair follicle inflammation through interferon-γ and interleukin-15 signaling pathways [13,14]. JAK inhibitors can blockade the interferon-γ and interleukin-15 signaling pathways, leading to the reverse of AA [15,16].

The efficacy and safety of BAB during AA treatment were studied and well-established by randomized, double-blind, placebo-controlled trials [17,18]. Treatment of AA with 2 mg and 4 mg of BAB exhibited adequate scalp hair coverage in 22% of 184 and 35% of 281 patients, respectively, compared to 5% of 189 patients with placebo doses. However, side effects associated with BAB treatment include the following: hyperlipidemia, URT and UT infections, folliculitis, Candida infections, neutropenia, and weight gain [19].

The pharmacokinetics parameters of BAB on oral administration seem to be dose-dependent. In this context, tmax can be achieved within an hour with an absolute bioavailability of 79%, plasma protein binding capacity of approximately 50%, and renal elimination of about 75%, followed by 20% dose elimination in feces [20]. BAB’s half-life was 8 h in healthy volunteers, and the lifespan of atopic dermatitis was 12.9 h [21,22]. BAB was classified as BCS III, representing high solubility and poor penetrability. However, BAB is practically insoluble. The solubility data of this drug, as reported in different solvents, are as follows: aqueous (0.357 mg/mL), ethanol (0.40 mg/mL), dimethyl sulfoxide (74 to 165.1 mg/mL), dimethyl formamide (50 mg/mL), and PEG-400 (72.4 mg/mL) [23,24,25,26]. Xenobiotic substances with poor solubility or permeability represent low bioavailability and therapeutic effects. In such instances, drug administration is higher for drugs with low bioavailability, which leads to toxicity [27].

Many studies have reported improved bioavailability and sustained drug release. Nanosuspension of BAB was developed using poly lactic-co-glycolic acid (PLGA) by leveraging the nanoprecipitation technique. This, in turn, showed 88% entrapment efficiency with sustained drug release of approximately 93% within 24 h [25]. Khaled et al. proposed using hybrid lipid PLGA nanoparticles to sustainably improve drug release, with enhanced bioavailability (2.92 times) compared to pure BAB [26]. More recently, BAB liposomes were developed with three different lipids and their combinations “L-α-phosphatidylcholine, a combination of lipids 1-palmitoyl-2-oleoyl-phosphatidylethanolamine and s1-Palmitoyl-2-oleoyl-sn-glycerol-3-phosphoglycerol”. The outcome of this study reflected improved drug flux in the cornea, and significant therapeutic effects appealed to its usage in Sjögren’s syndrome [24].

To the authors’ knowledge, a dosage form designed by three-dimensional (3D) printing technology has not yet been reported for BAB [28,29]. 3D printing is a versatile technique especially preferred in personalized medical care. Two processes are used for 3D printing—jetting and material. Moreover, there are about six manufacturing technologies for 3D printing. Fusion deposition modeling (FDM) or fused filament fabrication comprises a layer-by-layer material deposition through the hot nozzle and has the advantages of low maintenance and operation costs with a large build volume capacity [30]. Hot-melt extrusion loads the drug into the filament, which, in turn, can be passed through the nozzle to reach the 3D objects [31]. Suitable pharmaceutical-grade polymers used to develop the 3D-printed dosage forms are as follows: HPMC, HPC, Eudragit L, PVA, PEG-acetate, and Soluplus [32]. Polylactic acid (PLA) is one of the most extensively used polymers for 3D and 4D dosage designs and models due to its bio-derived and biodegradable properties [33,34]. It has the required tensile strength for FDM and melting point.

Furthermore, it can be recycled and is compostable. Globally, PLA is the most widely used polymer in 3D printing as per the report of most used polymers worldwide for 3D printing as of July 2018 [35]. Precursors of PLA are lactic acid extracted from corn and sugars. It is synthesized by direct polycondensation of lactic acid (LA), azeotropic dehydrative condensation, and ring-opening polymerization (ROP) of lactide. During the process, oligomers or prepolymers are catalytically converted into cyclic lactide, available in three forms—d lactic, L lactid, and meso lactide. The final stage comprises the ring-opening polymerization of lactides to obtain PLA [36,37,38,39,40,41,42].

Liu et al. reported the controlled release behavior of ciprofloxacin-coated PLA surgical sutures, which reflect PLA biodegradation in the human body with drug release at the infectious wound sites, thereby accelerating the wound healing process [43]. Kukkonen et al. produced glibenclamide-encapsulated PLA 3D-printed tablets by leveraging FDM. Increments increased the drug release from the filament in the drug loading solution concentration, including arginine—a solubility enhancer for the glibenclamide drug [44].

The current investigation aimed to load BAB in the PLA filament and study the effects of 2 and 4% BAB on filament loading capacity. Furthermore, this study characterized the effects of solvents on the PLA and soaking effects. The BAB-loaded PLA 3D tablets were fabricated by an FDM printer using PEG 200 as a solubilizer. Furthermore, the physicochemical and pharmaceutical parameters were characterized for the fabricated 3D pills.

## 2. Materials and Methods

Materials: BAB was purchased from Meso ChemTech Co., Ltd., Beijing, China. The PLA filament was purchased from FILO3DPRO, made in Holland. The solvents—ethyl acetate, dichloromethane, and DMSO—were purchased from a laboratory reagent (in India). Moreover, polyethylene glycol 400 was procured from Loba Chemie (Mumbai, India), whereas acetone, ethanol, and methanol were purchased from Sigma-Aldrich, St. Louise, MO, USA. All other chemicals used were of analytical grade and were used as supplied.

### 2.1. Selection of Solvent Blend by Hansen’s Parameters and Drug Solubility

A combination of solvents suitable for PLA was selected by changing the ratios to obtain the lowered Hansen’s solubility parameters (HSP) distance Ra value. An open-access HSP parameter calculator was utilized considering the dispersion (atomic) force (δ_D_), permanent molecular bipolar force (δ_P_), and hydrogen bonding (δ_H_) of the solvents to estimate the miscibility and interactive forces between the different target materials [45,46,47,48]. Some solvents suitable for pharmaceutical purposes and target materials (PLA) were checked for Ra value using the online HSP software [49]. Solvents such as methyl acetate (MeAt), dimethyl sulfoxide (DMSO), acetone (ACTN), ethyl acetate (EA), ethanol (EtOH), 2-propanol (2-PrOH), and water (H_2_0) were studied to calculate HSP distance (Ra value). Moreover, the combination of the solvents was also considered to secure the most miscible solvent blend suitable for PLA [50]. Based on the acceptable filament physical characteristics, ACTN and EtOH were selected, and the ratio was varied to get the lowered Ra value. The ratio of ACTN and EtOH selected for 5 gm of PLA was 27.8:18.2. Therefore, a blend of solvent was prepared by mixing 13.97 mL:9.1 mL (ACTN and EtOH).

A solubility study of BAB was performed by dispersing (5 mg) drug into (5 mL) solvents (ACTN and EtOH), surfactants (PEG-200 and Tween80), and water. The dispersions were added to screw-capped bottles and placed into the mechanical stirrer water bath for 24 h. Excess BAB was added if the solvent or surfactant became saturated with the drug, and the experiment continued after 48 h. Herein, aliquots were derived from each sample and analyzed for drug concentration using a spectrophotometer—Jasco V640 (made in Tokyo, Japan) at λ_max_-251 nm [51,52].

### 2.2. Soaking of PLA Filaments and Swelling Test

About 10-mm PLA filament equivalent (30 mg) was cut into pieces and placed in wide mouth autoclavable clear neutral glass capped bottle “McCartney universal” with organic solvents methyl acetate (MeAt), dichloromethane (DCM), acetone (ACTN), ethyl acetate (EA), polyethylene glycol (PEG 200), and ethanol (EtOH). All these bottles were placed in the mechanical shaker (water bath) under front-back movement maintained at 40 °C. After 24 h, the filaments were observed for their physical properties and integrality, and only the filament with the ensemble was removed. The selected filament was blotted with cotton tissue paper and placed on the analytical balance weighing pan (model AUY220, Shimadzu Scientific Instruments, made in Kyoto, Japan). Subsequently, the filament swelling percentage was calculated using the following equation [44,45]:

$$Swelling\;index\;\left(\%\right)=\frac{Weight\;of\;solvent\;soaked\;filmanet-Weight\;of\;dry\;filamnt\;}{Weight\;of\;dry\;filmanet}\times 100$$

In addition, a swelling study was performed in the blend solvents by taking a filament (95 mm) weighing 292.5 mg, and placing it into 5 mL of ACTN and EtOH in the ratio 27.8:18:2, which was added into a glass-capped bottle placed in the mechanical shaker (water bath) at 40 °C. Again, SI was calculated, as mentioned in this section [51,52].

### 2.3. Encapsulation of BAB in PLA Filament

Pre-fabrication studies and trials were conducted to select the blank PLA unprocessed filament used to design 3D pill weighing. Herein, the filament (blank) was sufficient to fabricate 30 3D pills measured by weight and length. Filament PLA, 200 cm weighing (6.15 g) was used and 2 gm and 4 gm of the drug along with PEG 400 (1:1 *w*/*w*) of the drug and PEG 400 were dispersed into the solvent blend (7:3 *v*/*v*), 70 mL ACTN:30 mL EtOH. The filaments in the drug solution were allowed to be soaked at 37 °C with mechanical agitation. After 24 h of drug diffusion into the filament, the adsorbed drug was scrubbed and analyzed for drug analysis by spectroscopy (UV-spectrophotometer, Jasco Model-640, made in Japan). Set λ_max_ was 251 nm using ACTN:EtOH:PEG 400 solution as blank [44,53,54].

### 2.4. BAB 3D Pill Printing by FDM

BAB 3D pills were printed using an FDM-based 3D printer (ET Model X1, made in China). The pill’s geometry was designed using computer-aided software Autodesk Fusion 360. The 3D pills were designed to be round with a hole in the center. The hole in the pill’s center enhances the drug release due to the increased surface area. The dimensions of the pill are 12 mm in diameter and 4 mm in thickness, with a 6 mm inner hole diameter. The pill design was saved as a Standard Triangle Language (STL) file describing the design’s surface geometry. Subsequently, this STL file was transferred to a 3D printer software, which converted this file to a printer-readable G-code (geometric code for computer numerical control). The G-code for the designed 3D pill was formed by using a 3D printer slicer software Repetier-Host V2.2.4 (with Slic3r Slicer). This G-code included the slicing details for the printer’s design and printing parameter settings. The layer thickness, shell thickness, and initial layer thickness were set to 0.1, 1.2, and 0.3 mm, respectively. The infill density was 100% with raft support. The printing, travel, and bottom speeds were set to 50, 80, and 20 mm/s, respectively. The completely dried drug-loaded filament (BAB-loaded PLA filament) was fed into the extruder of the 3D printer, wherein the filament melted and extruded through the nozzle over a build plate and formed the designed 3D pill in a layer-by-layer pattern. The diameter of the loaded PLA filament was 1.75 mm, and the extruder nozzle size was 0.4 mm. The nozzle temperature was set to 210 °C, and the bed temperature was set to 40 °C. After printing, the 3D-printed pill was removed from the printer bed, separated from raft support, and subjected to further characterization [31,33,45].

### 2.5. Fourier Transform Spectroscopy

FTIR spectrums of BAB, unprocessed PLA filament, BAB loaded in PLA filaments, and 3D pills (3DP1 and 3DP2) were captured by placing the sample on the radiation window and fixed with a screw or bolt. FT-IR Spectrometer iD5 ATR diamond Nicolet iS 5 (Thermo Science, Waltham, MA, USA) was used. The samples were scanned in the wavenumber range between 4000 and 400 cm^−1^. The FTIR spectrums with intense peaks were noted and compared, and the functional groups’ peaks were then interpreted to confirm the compatibility of the drug (BAB) and target polymers (PLA) [55].

### 2.6. Characterization of the 3D Printed Pill

#### 2.6.1. Pills’ Weight Variation, Density, and Hardness Test

In total, 20 3D-printed pills were randomly selected, and the individual weight and mean weight of all 20 pills were determined. The quality control test of percent weight variation was calculated using the following equation [55]:

$$Weight\;variation\;\left(\%\right)=\frac{Individual\;weight-Average\;weight}{Average\;weight}\times 100$$

$$\rho =\frac{W}{{\left(\frac{D}{2}\right)}^{2}\;\times \;\pi \;\times \;H}$$

The pills’ density was also calculated by measuring their height (*H*), diameter (*D*), and weight (*W*) using a digital Vernier caliper and weighing balance, respectively. The constant (π) and density (*ρ*) were then calculated and reported. The calculated geometric densities indicated the intactness and absence of pores in the solid dosage of the pills.

#### 2.6.2. Assay of 3D Pills

3DP1 and 3DP2 pills fabricated with 0.32% and 0.61% drug-encapsulated filaments were first dissolved into 2 mL organic solvent (DCM) and 8 mL of solvent blend ACTN—EtOH. The dissolved pill solution was then passed through a 0.45 µm syringe septum and analyzed, as mentioned, in the solubility section using UV-spectrophotometer (JASCO, V-650, made in Japan) at 251 nm [25,26]. The percentage of the drug was then calculated and expected to be within the pharmacopeia limits, that is, between 95 and 105%.

#### 2.6.3. Differential Scanning Calorimetry

Samples under investigation included unprocessed PLA, pure BAB, BAB-loaded filaments (2% and 4%), and 3D pills (3DP1 and 3DP2), which were crimped individually into the hemispherical aluminum pan (approximately 5 mg sample weight). The compressed sample pan was placed in the heating chamber beside the reference (empty pan). Thermogravimetric analysis (SINCO, made in Republic of Korea) was performed by passing N_2_ gas 20 mL/min and heating at 20 °C from 30 to 300 °C [56]. Subsequently, the exothermic peaks of all samples were noted, collaged, and interpreted using the DSC N650 software.

### 2.7. In Vitro Dissolution and Release Kinetics

A dissolution study was performed using USP–II dissolution apparatus, wherein the basket was filled with 0.1 N HCl (500 mL) maintained at 37 ± 0.5 °C. A released study was performed for 3D pills (3DP1 and 3DP2). Aliquots (2 mL) were withdrawn at predetermined intervals and replaced with the same fresh medium. In the case of UV-spectroscopy-analyzed samples, the concentration was calculated, and the drug profile percentage was plotted against time [57].

The release data was then computed into the kinetics equations, and the mechanism of drug release was postulated. The kinetics equation used to calculate the release mechanism is as follows [26]:

Zero order rate kinetics

$$Q_{t}-Q_{0}+K_{0}t$$

First-order rate kinetics

$$Log\;Q_{t}=\mathrm{Log}\;Q_{0}-\frac{K_{1}t}{2.303}$$

Higuchi matrix model

$$Q_{t}=K_{H}\;t^{1/2}$$

Korsmeyer–Peppas model

$$F_{t}=[Q_{t}\;/Q]=K_{P}\;t^{n}$$

where Q_t_ is the amount of drug released in time t, Q_0_ is the initial concentration at time t, and Ft is the fraction of the drug released at time t. K_0_, K_1_, K_P_, and K_H_ indicate the zero, first order, Higuchi rate constants, and Korsmeyer–Peppas model constant. However, exponent (*n*) has different representations based on the number—≤0.45, 0.45 < *n* < 0.89, *n* = 0.89, and *n* > 0.89. This indicates Fickian diffusion (Case I diffusional), Anomalous (non-Fickian) diffusion, Zero order release (Case II transport), and Super case II transport, respectively.

### 2.8. Stability Testing

3D printed pills (3DP1 and 3DP2) were subjected to accelerated stability testing at 25 °C/60% RH or 40 °C/75% RH for one month. After a month, the samples were analyzed for drug release, and a similarity index was calculated to confirm the impact of environmental factors on release during the storage conditions (shelf life) [58]. The drug release profiles were computed in the equation before and after the test as per SUPAC guidelines.

$$f_{2}=50\times \log\{[1+(1/n)\;\sum_{t-1}^{n}{\left(Rt-Tt\right)}^{2}{]}^{-0.5}\times 100\}$$

where *f*_2_ is the similarity factor, *n* indicates the dissolution time, and *R_t_* and *T_t_* are the reference and test dissolution points at time *t*.

### 2.9. Statistical Analysis and Applied Computer Programs

All data presented herein are the mean ± standard deviation (SD). Statistical analysis was performed in Microsoft Excel 2016. Computer-aided design software Autodesk Fusion 360 was used to design 3D pills and acquire standard tessellation language (STL) files.

## 3. Results and Discussion

### 3.1. Selection of Solvent Blend by Hansen’s Parameters and Drug Solubility

**Figure 1:**
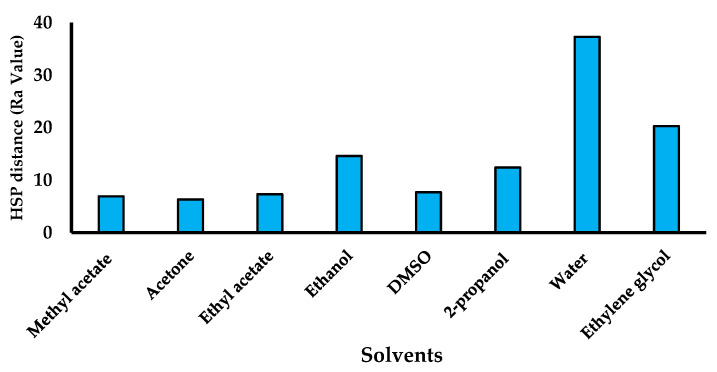
HSB parameters’ study with Ra value for the selected solvents.

HSP based on cohesive energy, which comprises dispersion (atomic) force (δ_D_), permanent molecular bipolar force (δ_P_), and hydrogen bonding (δ_H_) distance Ra value of the solvents, has been depicted in Figure 3. The order of solvents can be given based on the Ra value as follows: H_2_O > EtGlycol > EtOH > 2-PrOH > DMSO > EA > MeAt > ACTN. The soaking of filament in the selected solvent blend is depicted in Figure 1.

BAB solubility was 0.28 mg/mL, 0.46 mg/mL, 0.38 mg/mL, 68.7 mg/mL, and 0.52 mg/mL for water, ACTN, EtOH, PEG-400, and Tween80, respectively. Lowered Ra value is the best solvent for PLA. If the Ra value is small, as indicated by the HSPs for the two entities, they are considered similar and have more affinity for each other. Moreover, two solvent blends were also considered based on the Ra value and filament intactness in the solvent. HSP parameters then calculated the ratio of ACTN, EtOH, and PLA. The ratio was 15.5, 10.4, and 7.0 with a Ra value of 6.3, which is relatively less and is considered the best solvent mixture for PLA. If the HSP value of the two solvents is the same or close, then they are miscible with each other, expressed as “like dissolves like”. The solvent blend and appropriate drug solubilizer showed compatibility and diffusion capability, and were henceforth selected for drug encapsulation.

### 3.2. Soaking of PLA Filaments and Swelling Test

**Figure 2:**
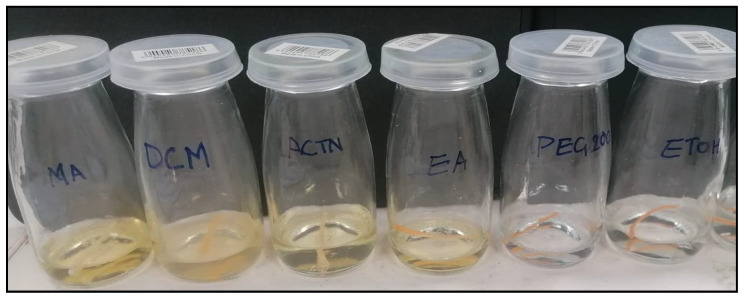
Swelling index study of PLA in the selected organic solvents.

**Figure 3:**
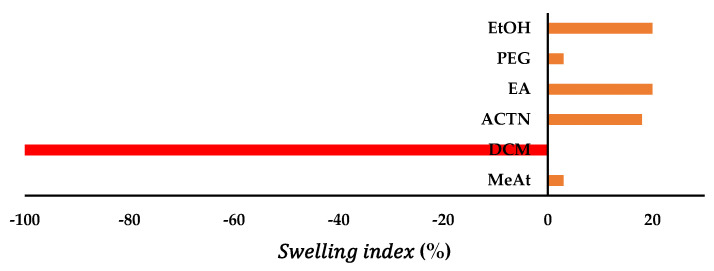
Swelling index percentage of PLA in the selected organic solvents.

Based on the swelling index solvent blend composed of acetone—ethanol (27.8:18:2) and PEG 400 in 1:1 drug—PEG 400 solubilizer ratios were selected, and the filament SI% after 24 h was found to be 7.77. This solvent system was used for the soaking and drug encapsulation through PLA filament. The filament was soaked into the solvents, and weights of the swelling index (SI) of MeAt, DCM, ACTN, EA, PEG, and EtOH were found to be 3.12%, −100%, 7.85%, 20.12%, 1.08%, and 2.08%. The filament was slightly swelled in PEG 400 and EtOH and bulging in ACTN, MeAT, and EA. However, DCM completely dissolved the filament (Figure 2 and Figure 3). Therefore, DCM is unsuitable for soaking purposes, especially with PLA.

### 3.3. Encapsulation of BAB in PLA Filament

**Figure 4:**
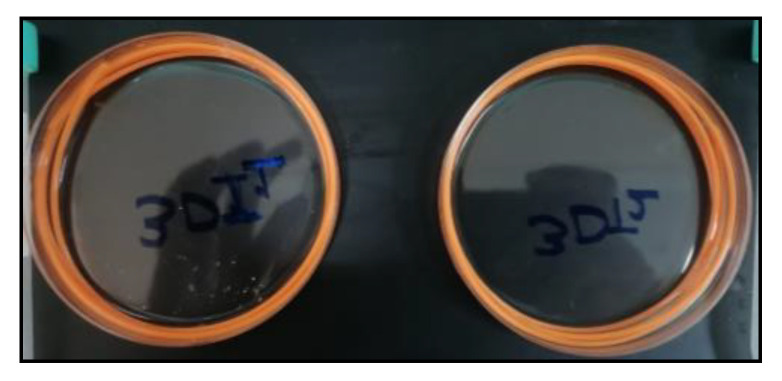
Encapsulation of BAB in the PLA unprocessed filament (3DP1 and 3DP2).

Blank filament soaked in 2% and 4% BAB involved PEG 400 (1:1) dispersion dissolved in a solvent blend of ACTN and EtOH (27.8:18.2). This approach showed the drug encapsulation of 0.32% and 0.61% for 3DP1 and 3DP2, respectively. The drug encapsulated into the filaments was then used as the additive material to print 3D pills, as shown in Figure 4.

### 3.4. BAB 3D Pills Printing by FDM

**Figure 5:**
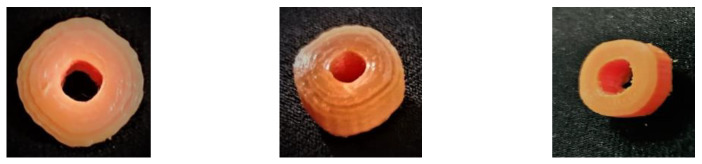
3D pills fabricated by FDM using PLA encapsulated with BAB.

A 3D CAD model designed the 3D pill. The STL file was then changed to the G-code file (Geometric code for computer numerical control), saved on a microSDXC card, and placed into the printer, considering the slicing and printing parameters described in Table 1. Subsequently, a drug-encapsulated filament was extruded, and 3D pills were scrapped from the printer stage and preserved for further characterization. The pill was designed as a doughnut to increase the surface area, as shown in Figure 5. Therefore, the dissolution solvent contact with the pill increased and catalyzed drug release from the matrix.

### 3.5. Fourier Transform Spectroscopy

**Figure 6:**
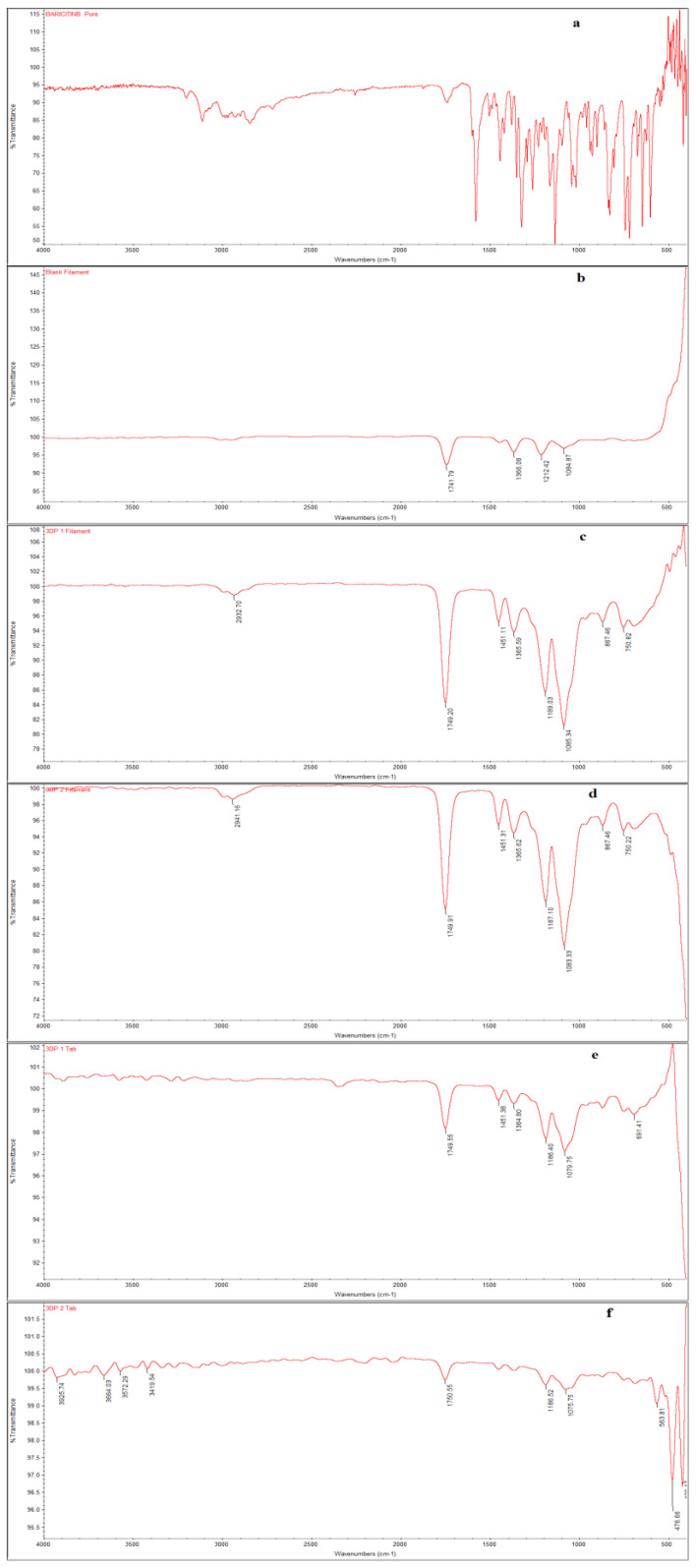
FTIR spectrums of (**a**) BAB, (**b**) PLA, (**c**) 3DP1, (**d**) 3DP2 filaments, and (**e**,**f**) for 3D pills fused with BAB.

BAB in the pure drug spectrum exhibits the characteristics peaks at 3345 cm^−1^, 3012 cm^−1^, 2654 cm^−1^, and 2322 cm^−1^ for the N-H stretching, aromatic = C–H stretching, –C–H stretching, and –C=N stretching. Moreover, characteristics peaks were observed at 1579.7 cm^−1^, 1442.96 cm^−1^, 1251.09 cm^−1^, 1322.80 cm^−1^, 1291.65 cm^−1^, 1261.41 cm^−1^, 1135.08 cm^−1^, 926.77 cm^−1^, 673.06 cm^−1^, and 601.07 cm^−1^, identical to the results in the BAB drug (Figure 6a). However, PLA showed prominent functional group peaks at 3100.15, 1741.79, 1366.09, 1212.42, and 1084.87 cm^−1^ for -OH (COOH), –CH3(S), –C=O, –C–O (COOH)–OH, –CH3(B) functional groups, respectively (Figure 6b). The characteristic peaks of drug and polymer (PLA) were identical in the 3DP1 and 3DP2 filaments, suggesting that BAB was successfully encapsulated within the PLA filament used for pill fabrications (Figure 6c,d). Moreover, the FTIR spectrums of 3D pills fused with BAB shown in (Figure 6e,f) also exhibit the identical peaks of drug and polymer accords compatibility of PLA with BAB. Decreased intensities of the peaks could be due to the heating temperature used in the fabrication of pills by which the molecules will occupy the excited vibrational states.

### 3.6. Characterization of the 3D-Printed Pills

#### 3.6.1. Pills’ Weight Variation, Density, and Hardness Test

All the FDM printed pills of both strengths, namely 3DP1 and 3DP2, were assessed for weight variation. The results ranged from −1.39% to 0.93% and −0.84% to 1.29%, respectively. As per USP standards for pills weighing from 130 to 324 mg maximum allowed % difference is 7.5%, stranded on which both the pills fabricated with BAB in PLA (3DP1 and 3DP2) passed the weight variation test. In this study, BAB loaded is less than 50% of the PLA weight. This test does not signify uniform drug distribution in the polymer. However, this test would be satisfactory for the solid dosage form on a drug substance of more than 50% of the dosage form unit.

The density of pills (3DP1 and 3DP2) was found to be 0.971 mg/mm^2^ and 0.993 mg/mm^2^, ensuring that fabricated pills do not have any void space or air space, indicating the failure of printing of solid mass. 3DP2 has relatively solid mass and excellent density and firmness compared to 3DP1.

#### 3.6.2. Assay of the 3D Pills

BAB encapsulated into 3DP1 and 3DP2 pills have shown the drug content to be 0.3 ± 0.01% and 0.6 ± 0.02%, respectively. A decrease in the drug concentration in the 3D pills compared to the drug-loaded filament could be attributed to the degradation and fusing of drug within the polymer matrix, which retards the drug dissolutions in the solvent, thereby indicating less pill content.

#### 3.6.3. Differential Scanning Calorimetry

**Figure 7:**
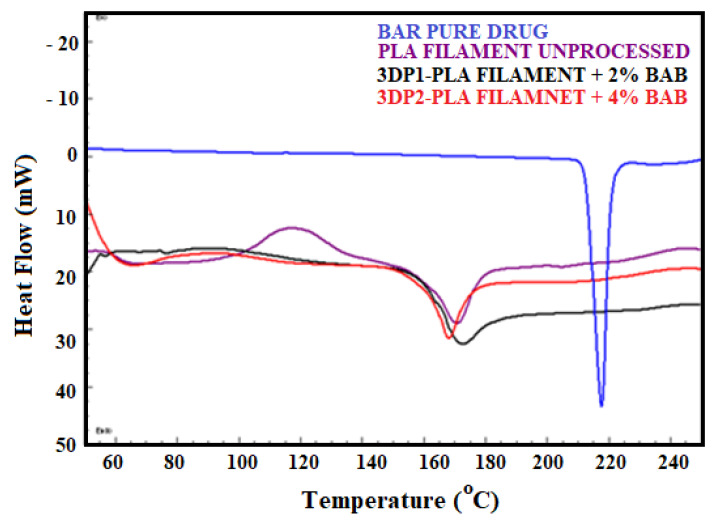
DSC thermograms of BAB, PLA, 3DP1, and 3DP2 pills.

The thermogram of the unprocessed PLA filament showed exothermic peaks at 110.5 °C and endothermic peaks at 170.5 °C. Pure BAB represented a sharp endothermic peak at 217.5 °C. Slight shifts in the melting peaks of PLA observed in 3DP1 and 3DP2 pills (Figure 7) could be attributed to increased or decreased L-isomer and D-isomer content. Moreover, it has been reported that PLA has two forms—the α-form (orthorhombic, pseudo-orthorhombic, or pseudo-hexagonal) and the β-form (trigonal or orthorhombic). The α-form melts at higher temperatures, whereas the β-form melts at lower.

Modification in the DSC peaks of unprocessed filament can be attributed to the additives, extrusion, and processing parameters used to fabricate the filaments. However, the cause is beyond the scope of this study.

However, printed pills BAB encapsulated 3D1 and 3D2 exhibit endothermic melting peaks at 170 and 165.1 °C. These shifts and modifications in the thermograms in 3D printed pills could be attributed to the thermal cycling due to the fused deposition modeling. The cold crystallization in the printed pills is due to the phase changes in the materials used (PLA filaments) above the glass transition temperature.

The exothermic peak was absent in the fabricated pills. However, the identical drug peak is insignificant in the DSC thermograms, assuring that the drug gets completely fused within the PLA pill matrix. As indicated by the MP peak, the disappearance of the crystal drug could be converted to an amorphous form due to the FDM processing and solvent effects.

### 3.7. In Vitro Dissolution and Release Kinetics

**Figure 8:**
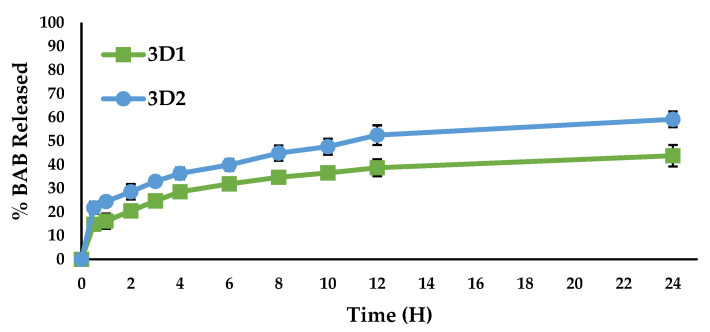
Percentage drug release—time plot for 3DP1 and 3DP2.

Drug release in studies with 3DP1 and 3DP2 pills composed of 22% and 37% BAB encapsulation in the filament was used to design the BAB oral pills. Both pills observed slight drug release bust effects due to the drug being completely encapsulated during the FDM heating process within the PLA. However, within an hour, 16.13 ± 1.45% and 24.37 ± 3.24% drugs were released, and the release noted were 44.76 ± 3.34%, 59.14.14 ± 4.54% up to 24 h from 3D1 and 3D2 pills, respectively. This pattern of release is desired to achieve the onset of action followed by the sustained effects of the drug for a prolonged period (Figure 8).

Fabricated 3D printed pills are expected to stimulate drug release by following any one or combination of these mechanisms—amorphous PLA–BAB over the designed pill, PLA transformed to semi-crystalline state or swelled, forming the water-filled microspores through which drug diffuses, and/or drug precipitation as crystal in contact with the dissolution medium, resulting in dissolution. The release mechanism was also calculated by computing the data into the kinetic equations.

The regression number of zero, first, Higuchi, and Korsmeyer–Peppas for 3D1 and 3D2 was exhibited to be 0.6763, 0.7495, 0.9176, and 0.9753, respectively, and 0.6975, 0.8171, 0.9226, and 0.9916, respectively. Both 3D1 and 3D2 followed Korsmeyer–Peppas’ order of drug release, representing the medium diffusion into the matrix, swelling, and slow matrix erosion. The erosion of PLA in human tissue has been well reported and can be gradually degraded into normal sugar metabolite and lactic acid (LA), followed by carbon dioxide and water. Based on the release exponent *n* value, the release mechanism is indicated to be Fickian transport. The sustained release of BAB is desired for managing AA and reducing the drug’s toxicity. The administration of BAB (Olumiant^®^) in AA therapy causes side effects, such as increased cholesterol levels, muscle enzyme levels, lower RBC and WBC, and increased weight, in some people.

### 3.8. Stability Testing

As per the stability study, the *f*_2_ value of the pills was calculated, and the result was within the range of 51 ± 1.08, indicating that the release profiles are the same before and after the environmental factor exposure.

## 4. Conclusions

In this study, the 3D pills were fabricated by FDM technology. The solvent combination was selected based on the Ra value and soaking power. PEG 400 was also included in the drug loading process, as it acts as a solubilizer and facilitates and improves the drug diffusion inside the filament. The 3DP1 and 3DP2 pills showed an acceptable % of weight variation and drug content as per the compendia standards. FTIR and DSC studies highlight that pills 3DP1 and 3DP2 have BAB compatibility with PLA and the amorphous state of the drug in the PLA-designed mass. Drug release from both pills progressively increased with increments in the drug load in the filament and drug solution added for the soaking process. The 3D printed pills of BAB were fabricated with bio-derived-biodegradable PLA with a low processing cost and proved to be practicable in the lab set-up. A further pilot study of this prototype will help facilitate the personalized medicine and AA treatment of patients in clinical settings.

## Figures and Tables

**Table 1:** Slicing and printing parameters for the 3D pill.

STL File	Slicing Parameters		Printing Parameters	
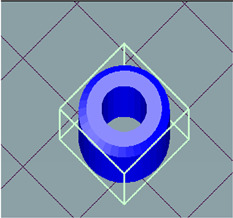 **3D pill design**	Layer thickness	0.1 mm	Printing temperature	210 °C
	Shell thickness	1.2 mm	Bed temperature	40 °C
	Initial layer thickness	0.3 mm	Printing speed	50 mm/s
	Infill density	100%	Travel speed	80 mm/s
	Adhesion	*Raft*	Bottom speed	20 mm/s

STL—stereolithography.

## Data Availability

Not applicable.
